# Supplementary material for: Factors associated with modern contraceptive use among men in Pakistan: Evidence from Pakistan demographic and health survey 2017-18
Source: PLoS One. 2022 Sep 1;17(9):e0273907. doi: 10.1371/journal.pone.0273907 (PMC9436105; doi:10.1371/journal.pone.0273907)
Supplement: S1 Table — (DOCX) [file pone.0273907.s002.docx]

**Table 1. Findings of collinearity diagnostics (tolerance statistics and variance inflation factor).**

| **Covariate** | **Tolerance statistics** | **Variance inflation factor (VIF)** |
| --- | --- | --- |
| **Age** | 0.81 | 1.22 |
| **Region** | 0.92 | 1.08 |
| **Residence** | 0.73 | 1.35 |
| **Education** | 0.73 | 1.35 |
| **Wealth index** | 0.58 | 1.70 |
| **Currently working** | 0.98 | 1.01 |
| **Contraception is women’s business** | 0.60 | 1.65 |
| **Use of contraception makes women promiscuous** | 0.61 | 1.62 |
| **Discussed family planning with a health worker** | 0.93 | 1.06 |
| **Fertility preference** | 0.81 | 1.22 |
