# Supplementary material for: Factors associated with modern contraceptive use among men in Pakistan: Evidence from Pakistan demographic and health survey 2017-18
Source: PLoS One. 2022 Sep 1;17(9):e0273907. doi: 10.1371/journal.pone.0273907 (PMC9436105; doi:10.1371/journal.pone.0273907)
Supplement: S2 Table — (DOCX) [file pone.0273907.s003.docx]

**Table 1. Descriptive table on various contraceptive methods.**

| **Contraceptive methods** | **frequency** |
| --- | --- |
| Not using | 2694 |
| Pills | 48 |
| IUDs | 40 |
| Injections | 64 |
| Male condoms | 394 |
| Male sterilization | 04 |
| Female sterilization | 136 |
| Periodic abstinance | 21 |
| Withdrawal | 199 |
| Implants/Norplants | 21 |
| Lactational amenorrhea | 05 |
| Emergency contraception | 01 |
| Standard days method | 01 |
