# Supplementary material for: Factors associated with modern contraceptive use among men in Pakistan: Evidence from Pakistan demographic and health survey 2017-18
Source: PLoS One. 2022 Sep 1;17(9):e0273907. doi: 10.1371/journal.pone.0273907 (PMC9436105; doi:10.1371/journal.pone.0273907)
Supplement: Attachment — Submitted filename: Review - PONE-D-21-23985 (1).pdf [file pone.0273907.s005.pdf]

Review of Manuscript Number PONE-D-21-23985

The question of fertility plays a vital role in many countries' economic development and health objectives, including Pakistan. It is, therefore, an important area for investigation and the use of large datasets such as the demographic and health survey (PDHS). I appreciate the authors' curiosity and diligence in investigating this area. Hopefully, my comments can be used to enhance the work.

Introduction

- On page 03, line 51, the reference to fertility reduction as being connected to improvements in health and economic activity is a bit vague. Are you referencing individuals or the state of the country as a whole? Also, it is a bit of a nuanced argument that would require some expansion. However, your study seems more concerned with reaching the male population and less of the significant picture objectives, perhaps focusing on the male perspective. Why have males historically been left out of the conversation on family planning? Why has it taken so long to recognize the importance of reaching out to males in that space? Why have previous efforts tended to focus on women?
- On page 4, Line 73 -Line 74, you refer to the United Nations International Conference on Population and Development. That was more than a generation ago, and there have been many iterations of global, regional, and country-specific policies and objectives that have addressed some of the questions you are investigating in this paper. Perhaps you should reference something more recent (e.g., the Millennium Development Goals, followed by the SDGs). Even the UN ICPD had a 25th reunion (i.e., the Nairobi Summit). How have such global structures framed the issue of fertility and contraceptive use behaviors?

Methods

- The methods read as straightforward and well-executed. I have nothing to add to this section except for the choice of the conceptual framework. Why was that framework chosen? What is the justification for choosing to look at the variables of interest through that lens? Several conceptual frameworks have been used over the years to explain contraceptive use and family planning behaviors (e.g., USAID's Conceptual Framework and the Women's and girls' empowerment in sexual and reproductive health (WGE-SRH) framework). Why did you eschew other frameworks in favor of this one?
- As a note, Figure 01 is blurry, and I think you should find a better image that is clearer and easier to read.

Results

1. Be careful of the language you employ in describing your results. For example, in the results section, you use the terms likelihood and odds interchangeably. Though they are used to mean the same thing in colloquial usage, "odds"

references a particular relationship between the ratio of probabilities (see pg. 10, Line 171 compared to Line 175).
- Also, be sure to state that a finding is statistically significant and not just significant. Again, statistically significant as a technical term references something particular, while a finding being “significant” can have a more general meaning, such as being generally important or worth noting.

Discussion

The paper’s discussion section is an excellent place to explore your findings and their potential impact. As I read through this section, it mostly reads as a reiteration of the results section. Try exploring some of the following areas:
- Given that in the introduction, you mention that your findings may be used to inform policy, I was hoping the discussion would delve into some commentary about Pakistan’s policy regarding modern contraceptive use if there is one, and how findings can influence that. The fertility challenges have been documented for many decades now and I, as a reader, assume that there are some policies already in place. How do your findings impact those kinds of initiatives? Are men historically excluded from those policies? If men as a subgroup have factored into such initiatives, what do these findings mean?
- On pg. 13, Line 217 to Line 222, you note regional differences, particularly between Punjab and other regions. You theorize that it may be due to improve family planning programs. Is it possible to expand a bit on this? What kind of well-structured family planning programs in Punjab make that place more successful than other regions, and why have such policies not been diffused to other regions.
- Why do you relate your study findings to findings from places like Uganda and Congo? There is nothing wrong per se with that comparison; I don’t think. You could argue that Pakistan occupies a similar socio-economic bracket as those countries, and hence the way they deal with contraceptive use and family planning behaviors may have some bearing on Pakistan. However, neighboring countries in the subregion (e.g., India, Tajikistan, Afghanistan, and Iran) may offer more forthright comparators. I suggest looking through the literature.
