# Supplementary material for: Factors associated with modern contraceptive use among men in Pakistan: Evidence from Pakistan demographic and health survey 2017-18
Source: PLoS One. 2022 Sep 1;17(9):e0273907. doi: 10.1371/journal.pone.0273907 (PMC9436105; doi:10.1371/journal.pone.0273907)
Supplement: Attachment — Submitted filename: Review 02 - PONE-D-21-23985R1.pdf [file pone.0273907.s007.pdf]

Review 02: PONE-D-21-23985R1

Overall, great job in addressing my concerns from the first submission. I think the paper is ready to move forward. I would suggest a few more minor edits and things to consider. Please see my comments below.

Methods

You did not state the significance level in the Statistical Analysis section. Your audience needs to know the cutoff for statistical significance. I assume it is 0.05?

Results

Consider using two decimal places for the reported statistics even in your tables. Honestly, there is no need to use more than 2 in a study of this type. Furthermore, two decimal places improves readability.

I noticed this in Table 3, but consider creating a list of abbreviations/acronyms at the end of your article rather than using superscripts to designate the acronym. For example, FATA means, **Federally Administered Tribal Areas**. A list of abbreviations/acronyms would make for easy referencing.

Discussion

Line 247 - 248

You cannot, or perhaps should not, draw this type of conclusion. Yes, contraceptive use is strongly associated with the man's household socioeconomic bracket (measured by the Wealth Index). Will simply increasing household wealth improve contraceptive use? And who is to improve household income? Since you are looking at population-level data, I assume that will be the government? Are there no other confounding factors that mediate the relationship between wealth and contraceptive

use that may offer a better point for policy to address? Lastly, the Wealth Index used in the DHS program is a generalized measure that allows broad comparisons at the population level. Measures of wealth using income and expenditure would give more robust evidence for that association and thus would be better support for that conclusion, which could be a study for another time.

Your discussion section is missing an element. I think you are missing a paragraph on study limitations.

Conclusion

Line 286-287

What kind of policy do the authors recommend? Do you have any suggestions from the references that will help Pakistan achieve a contraceptive prevalence rate of 60% by 2030? I bring this up because you are the experts; you have studied the statistics and provided us with some answers, so from your analysis, what would be your policy recommendations? The conclusion offers you the opportunity to be a bit more specific and direct (this is just a suggestion, as it is fine as written).
