# Supplementary material for: Factors associated with modern contraceptive use among men in Pakistan: Evidence from Pakistan demographic and health survey 2017-18
Source: PLoS One. 2022 Sep 1;17(9):e0273907. doi: 10.1371/journal.pone.0273907 (PMC9436105; doi:10.1371/journal.pone.0273907)
Supplement: Attachment — Submitted filename: RESPONSE TO REVIEWRS.docx [file pone.0273907.s006.docx]

The Editor,

PLOS One.	Dated: 09/05/22

**Subject: Submission of revised version of the manuscript entitled: “Factors associated with modern contraceptive use among men in Pakistan: evidence from Pakistan demographic and health survey 2017-18”.**

Respected editor,

Thank you for providing us the opportunity to submit a revision of the manuscript titled “Factors associated with modern contraceptive use among men in Pakistan: evidence from Pakistan demographic and health survey 2017-18”. To PLOS One journal. We appreciate the effort invested by you and the reviewers to provide a valuable feedback on our study. The points highlighted by the reviewers have been very useful in enhancing the accuracy of our analysis. We have made a genuine effort to incorporate most of the changes suggested by the reviewers. We look forward to hearing from you and anticipate that our study will be considered for publication in PLOS one.

A “point to point” response to the reviewer’s comments is provided below.

**Reviewer no. 1**

- **Consider changing the word “determinants” to “factors associated with”, as DHS do not have a longitudinal design, thus cannot be utilized to study causality.**

The suggested change has been made.

- **The author says that U$ 55 are spent per woman, but it is not clear if it is a yearly rate.**

The suggested change has been made in line no. 61.

- **As much as the authors cite the relevant factors that matter for the utilization of modern contraceptive among men and bring some of these factors in the discussion (socioeconomic status, education, cultural beliefs, area of residence, religion and wrong perceptions about family planning), it would be interesting to hear more about these mechanisms up front. I would add a section containing a brief review of these factors, especially because testing these elements is the main goal of these analyses.**

Additional information from relevant literature explaining the mechanisms of various predictors on contraception has been added as per the reviewer’s suggestion (line 73-80).

- **The same goes for research studies “exploring the predictors of contraceptive use among couples have overlooked the role of men”. The authors should bring more of this literature and how their study contributes to this framework**

Additional information from relevant literature has been added as per the reviewer’s suggestion (line 93-94).

- **The following phrase is misplaced: “In agriculture based societies, men usually wish to have large number of children because they serve as a source of livelihood. This perception of men creates hinderance in the utilization of contraceptives by couples [18][19][20]”. This is an example of a mechanism and should be included in the section I suggest you add.**

I appreciate reviewer’s efforts and keen interest in my manuscript. The phrase has been repositioned (line no. 71-73).

- **The findings would also benefit from more background regarding the regions explored (Punjab, Sindh, KPKa, and so on). Why would I expect regions to differ regarding contraception use? Could levels of development and indicators of gender equity (such as female illiteracy rate) be used to explain some of these differences observed? What has the literature said of these places? You explain Punjab may have improved the structure of family planning services. I would like to hear more about it.**

Ministry of national health services regulation and coordination, Pakistan published a report that commented on the ranking of various provinces of Pakistan based on their conduciveness to contraception services. The report pointed out that because of better female literacy rate, women empowerment, better utilization of antenatal care services, contraception prevalence is better in Punjab. This information is incorporated in line no. 261-267.

- **In the end of the Background section, the authors highlight the importance of their findings to frame family planning programs considering the role of men as decision makers in the matters of family planning and reproductive health in patriarchal societies. I think this is gold and should be brought up again in the results with clear and stated recommendations.**

I am thankful to the reviewer for highlighting an important area of the introduction and its relevance to results and conclusion.

- **I also suggest you enrich your review by bringing information about factors associated with contraceptive use for Pakistan women**

The study intends to look for the factors associated with modern contraceptive use in men. Authors have tried to put together the most pertinent information from the literature. Additional information on women related factors may unduly prolong the introduction section and may appear disconnected from the rest of the literature.

- **The analysis was done carefully and the method is adequate for the research question. However, I think it is important to insert controls for parity and if men are sexually active.**

I appreciate the encouraging remarks of the reviewer. Data on “parity” and “if men are sexually active” could not be fetched from the dataset.

- **I also think it would be good to leave age as a continuous variable, unless you have reasons to believe those three age groups should remain separate**

Age was originally a categorical variable in dataset consisting of 5 years age groups. However it was recoded and divided into three classes for the ease of data interpretation.

- **I understand that calculating a Wealth Index using quintiles would automatically separate the observations into five groups with approximately the same number of respondents. So, these phrase is irrelevant: “Each wealth quintile had an approximately similar number of respondents”.**

The said phrase has been removed.

- **Regarding the logistic regression, I understood from the text that authors only used in the multivariate analysis the variables that had been found to be significant in the bivariate model. However, by looking at Table 3, all variables were included (indeed, they are all significant at the bivariate model). I would add a phrase explaining that they are all significant. If it is not what you did, please, clarify.**

All the variables except “employment status” were significant in bivariate analysis. Therefore, rest of the variables were recruited into multivariable model. The modification is present in line no. 162-164.

**Conclusion**

- **I think this manuscript would benefit from a Review as it has the potential to make a good contribution in its field.**

We are thankful to the editorial team and worthy reviewers for their valuable time and considering our study for peer review. Your comments are really valuable in improving the manuscript.

**Reviewer no. 2:**

The question of fertility plays a vital role in many countries’ economic development and health objectives, including Pakistan. It is, therefore, an important area for investigation and the use of large datasets such as the demographic and health survey (PDHS). I appreciate the authors’ curiosity and diligence in investigating this area. Hopefully, my comments can be used to enhance the work.

**Introduction**

- **On page 03, line 51, the reference to fertility reduction as being connected to improvements in health and economic activity is a bit vague. Are you referencing individuals or the state of the country as a whole? Also, it is a bit of a nuanced argument that would require some expansion.**

The said line has been eliminated.

**However, your study seems more concerned with reaching the male population and less of the significant picture objectives, perhaps focusing on the male perspective. Why have males historically been left out of the conversation on family planning? Why has it taken so long to recognize the importance of reaching out to males in that space? Why have previous efforts tended to focus on women?**

The generalized perception about “contraception being a women’s business” is largely responsible for keeping males from family planning programs target. The importance of male involvement particularly in patriarchal societies was recognized aa few decades back. International conference on population and development held in Cairo was among the initial historical landmark that endorsed the involvement of men in family planning programs. The relevant literature is stated in the Introduction section.

- **On page 4, Line 73 -Line 74, you refer to the United Nations International Conference on Population and Development. That was more than a generation ago, and there have been many iterations of global, regional, and country-specific policies and objectives that have addressed some of the questions you are investigating in this paper. Perhaps you should reference something more recent (e.g., the Millennium Development Goals, followed by the SDGs). Even the UN ICPD had a 25th reunion (i.e., the Nairobi Summit). how have such global structures framed the issue of fertility and contraceptive use behaviors?**

The ICPD held in Cairo was an important historical landmark that drew attention towards the role of men in family planning. Furthermore, Reference from international conference on population and development held in Nairobi has also been cited in the introduction (line no. 86-90).

**Methods**

- **The methods read as straightforward and well-executed. I have nothing to add to this section except for the choice of the conceptual framework. Why was that framework chosen? What is the justification for choosing to look at the variables of interest through that lens? Several conceptual frameworks have been used over the years to explain contraceptive use and family planning behaviors (e.g., USAID’s Conceptual Framework and the Women’s and girls’ empowerment in sexual and reproductive health (WGE-SRH) framework). Why did you eschew other frameworks in favor of this one?**

USAID conceptual framework is designed to assess the impact of family planning on various areas of women’s lives i.e. personal autonomy, health status, economic resources and acquisition of education etc. Whereas, our study intends to assess the factors that influence contraceptive uptake. Considering the research question and the available data, authors found the employed conceptual framework to be more pertinent.

- **As a note, Figure 01 is blurry, and I think you should find a better image that is clearer and easier to read.**

The image quality has been improved. Modified image is uploaded with the revised manuscript.

**Results**

- **Be careful of the language you employ in describing your results. For example, in the results section, you use the terms likelihood and odds interchangeably. Though they are used to mean the same thing in colloquial usage, “odds” references a particular relationship between the ratio of probabilities (see pg. 10, Line 171 compared to Line 175).**

The issue has been rightly raised. Appropriate changes have been made in the results section (line 203 and 210).

- **Also, be sure to state that a finding is statistically significant and not just significant. Again, statistically significant as a technical term references something particular, while a finding being “significant” can have a more general meaning, such as being generally important or worth noting.**

Authors understand the point raised by the worthy reviewer. Findings have been reported appropriately in this regard.

**Discussion**

The paper’s discussion section is an excellent place to explore your findings and their potential impact. As I read through this section, it mostly reads as a reiteration of the results section. Try exploring some of the following areas:

- **Given that in the introduction, you mention that your findings may be used to inform policy, I was hoping the discussion would delve into some commentary about Pakistan’s policy regarding modern contraceptive use if there is one, and how findings can influence that. The fertility challenges have been documented for many decades now and I, as a reader, assume that there are some policies already in place. How do your findings impact those kinds of initiatives? Are men historically excluded from those policies? If men as a subgroup have factored into such initiatives, what do these findings mean?**

A brief section has been added in the discussion section that highlights the policy in place for improvement of contraceptive prevalence in Pakistan. The section indicated that there is no clear policy in place that dictates the measures to encourage the involvement of men in family planning and decisions related to contraception (line no. 232-237).

- **On pg. 13, Line 217 to Line 222, you note regional differences, particularly between Punjab and other regions. You theorize that it may be due to improve family planning programs. Is it possible to expand a bit on this? What kind of well-structured family planning programs in Punjab make that place more successful than other regions, and why have such policies not been diffused to other regions.**

A brief explanation on regional variation in contraception usage has been added (line no. 261-267)

- **Why do you relate your study findings to findings from places like Uganda and Congo? There is nothing wrong per se with that comparison; I don’t think. You could argue that Pakistan occupies a similar socio-economic bracket as those countries, and hence the way they deal with contraceptive use and family planning behaviors may have some bearing on Pakistan. However, neighboring countries in the subregion (e.g., India, Tajikistan, Afghanistan, and Iran) may offer more forthright comparators. I suggest looking through the literature.**

The reviewer has rightly pointed out the rationale behind citing literature from places like Tanzania and Congo. Because of similar socioeconomic conditions, the findings from these regions may apply to the subcontinent as well.

**Reviewer no.3:**

Dear Editor,

Thank you for the opportunity to review the manuscript titled Determinants of modern contraceptive use among men in Pakistan: evidence from Pakistan demographic and health survey 2017-18.

Overall, the manuscript is fairly well written and has clear aims. It also focuses on a topic of deep interest to the reproductive health community and is backed by an extensive body of research. However, I find that the justification for the study and its relative contribution to the existing literature is very weak. As such, the unique contribution of these analysis is unclear to me. I think the authors can make a stronger case if they could point out which variables have not been examined, whether is consensus and variables that have inconsistent results. Please see below my specific comments that could further improve the manuscript.

**Introduction**

**Overall – Fairly clear and well written.**

- **There are multiple typos throughout the manuscript. Authors are suggested to proofread them carefully.**

The manuscript has been carefully proof-read. Any typo that was detected has been corrected and highlighted.

- **Consider reporting on how Pakistan compares in relation to regional estimates of modern contraceptive use.**

The estimates on contraceptive prevalence rate of India and Bangladesh has been added (line no. 56-57).

- **Need to define modern contraceptives.**

Modern contraceptives, being a dependent variable of the study, has been defined in methodology.

- **If effective strategies need to be country-specific, what was the rationale behind estimating prevalence? The rationale part needs to be strengthened.**

This study did not estimate any prevalence. With respect I would like to say that the rationale part was written carefully. I would like to refer to the comment of reviewer one that says:

**“In the end of the Background section, the authors highlight the importance of their findings to frame family planning programs considering the role of men as decision makers in the matters of family planning and reproductive health in patriarchal societies. I think this is gold and should be brought up again in the results with clear and stated recommendations.”**

**Methods**

- **Data sources and sampling techniques – could benefit from better organization structure – information seems randomly placed. Also, expand on stratification and provide a reference for a more elaborate description for the DHS sampling strategy.**

The sampling procedure used in PDHS 2017-18 has been added to the methodology section (line no. 109-117). A reference for the details of sampling collection technique used in the survey has been added (line no. 124-125). The information has been placed in a structured format. Information related to PDHS survey, data collection procedure is placed under the section of “data source”. This was followed by the definitions of variables used in the study. Finally. The statistical analysis used in this study was explained.

- **2. Sample –Provide a justification for your sample selection**

The sample consisted of 15-49 years old ever married men. This sample was used because the data on contraception was available only for the said bracket of men in PDHS dataset.

- **3. Please provide another Table and give how each of the independent variables were derived or recoded from the original dataset. This can either be in the manuscript or attached as a supplementary file.**

Only two variables (education and age) were recoded. The details on how the said variables were recoded from the original dataset have been incorporated into the manuscript (line no. 151-153)

- **4. Which sampling weight and id were used and was the weight normalized?**

Men’s sample weights were used from PDHS dataset. The ID of the weight variable in men’s PDHS dataset was “mv005”. Weights normalization was not carried out.

- **What informed the inclusion or selection of the independent variables?**

Available data and literature survey guided the recruitment of independent/predictor variables.

- **Please specify the model equation**

Upon reading various published studies in impact factor journals, it was found that most of the studies do not include the statistical equation in the methodology. As all of the tests are run in software, it is usually not necessary to have an in depth mathematical understanding of the statistical models to run these tests. However, if it is an essential requirement, authors will seek guidance from the statistician to help specify an equation for our model.

- **What informed the choice of the reference categories**

Previous studies were explored for this purpose. In addition, the reference categories were selected based on the hypothesis e.g. “residents of Punjab” and “educated men” were anticipated to have a higher modern contraception uptake. Thus, these were selected as reference categories to check how these categories differ relative to their counterparts.

- **8. Did the authors check for multi-collinearity, the results should be provided**

Yes. Multicollinearity was checked. The results have been added as a supplementary file named “S1 text”.

- **9. Please use the STROBE guidelines and present it as an appendix or a supplementary file**

STROBE guidelines were used during manuscript write-up. A strobe checklist has been attached as a supplementary file named “S2 text”.

**Results**

- **Consider specifying only the key findings of the study rather than listing all the determinants. Also, use the breakdown of the sentences rather than writing one long sentence that is hard to follow.**

Authors respect this concern of the reviewer. The results are already tailored to the key findings. Authors have gone through them in detail. None of them needs to be deleted. In addition, no sentence is long enough to interrupt the continuity of the reader.

- **The statistical analysis not been performed rigorously**

Critical comments are a great avenue for self reflection and learning. The statistical analysis was reviewed in detail. Reviewer 1 and 2 have also endorsed that data analysis is adequately carried out. Moreover, Authors have tried their level best to respond to the comments of each reviewer raised on statistical analysis.

**Discussion**

- **Authors started to compare and contrast study findings, which should have been followed after stating the key findings and justification for those findings.**

A summary of the study findings is added before discussing each of them separately (line no. 229-232). The justification for all the findings is appropriately provided. Wherever deficient, as indicated by the previous reviewers additional information has been incorporated to give a robust justification of the results. E.g. line 232-237 and line 261-267.

- **2. There is a repetition of most of the findings that are already stated under the results section.**

There is no reiteration of the findings per se in the discussion, however these are mentioned in order to compare them with the previous studies of similar kind.

- **3. Justification for all the discordant results are presented same i.e., due to differences in sample size, study design, setting, and study population. This needs to be study specific rather than a mere generalization.**

There is no such recurring justification based on study population, design or sample size in the discussion section. Moreover, various modifications have done in the discussion section which have led to improvements in the justification of reported findings.

**Conclusion**

- **Clearly and concisely state the conclusions of the study in relation to the key question it sought to answer and the contribution that the paper would make.**

The conclusion section has been enriched with the policy recommendation and the importance of its potential to contribute to the improvement of contraception prevalence has been added (line 286-290).

- **2. The conclusion is well presented. However, the policy implications are not well discussed. The authors can consider beefing them up.**

I appreciate the reviewer’s encouraging remark. The policy implication has been added to the conclusion (line 286-290).
